# Use of Extracellular Monomeric Ubiquitin as a Therapeutic Option for Major Depressive Disorder

**DOI:** 10.3390/ph17070841

**Published:** 2024-06-27

**Authors:** José Luis Maldonado-García, Lissette Haydee García-Mena, Danelia Mendieta-Cabrera, Gilberto Pérez-Sánchez, Enrique Becerril-Villanueva, Samantha Alvarez-Herrera, Toni Homberg, Luis Vallejo-Castillo, Sonia Mayra Pérez-Tapia, Martha C. Moreno-Lafont, Daniel Ortuño-Sahagún, Lenin Pavón

**Affiliations:** 1 Departamento de Inmunología, Escuela Nacional de Ciencias Biológicas, Instituto Politécnico Nacional, Mexico City 11340, Mexico; joselmgarci@comunidad.unam.mx (J.L.M.-G.); sperezt@ipn.mx (S.M.P.-T.); 2 Departamento de Bioquímica, Facultad de Medicina, Universidad Nacional Autónoma de México, Mexico City 04360, Mexico; 3 Laboratorio de Psicoinmunología, Dirección de Investigaciones en Neurociencias, Instituto Nacional de Psiquiatría Ramón de la Fuente Muñiz, Mexico City 14370, Mexico; gilberto.perez.sanchez@inprf.gob.mx (G.P.-S.); lusenbeve@inprf.gob.mx (E.B.-V.); dra.alvarezherrera@gmail.com (S.A.-H.); 4 Departamento de Salud Digital, Facultad de Medicina, Universidad Nacional Autónoma de México, Mexico City 04360, Mexico; lissettegarcia@comunidad.unam.mx; 5 Servicios Clínicos, Instituto Nacional de Psiquiatría Ramón de la Fuente Muñiz, Ciudad de México 14370, Mexico; danemend@hotmail.com; 6 Unidad de Desarrollo e Investigación en Bioterapéuticos (UDIBI), Escuela Nacional de Ciencias Biológicas, Instituto Politécnico Nacional, Mexico City 11340, Mexico; investigacion.clinica@useic.com.mx (T.H.); lavallejos@ipn.mx (L.V.-C.); 7 Laboratorio Nacional Para Servicios Especializados de Investigación, Desarrollo e Innovación (I+D+i) Para Farmoquímicos y Biotecnológicos, LANSEIDI-FarBiotec-CONACyT, Mexico City 11340, Mexico; 8 Instituto de Investigación en Ciencias Biomédicas (IICB), CUCS, Universidad de Guadalajara, Jalisco 44340, Mexico; daniel.ortuno@academicos.udg.mx

**Keywords:** major depressive disorder, immunomodulator, inflammation, extracellular monomeric ubiquitin, therapeutic options based in small proteins

## Abstract

Major depressive disorder (MDD) is a mood disorder that has become a global health emergency according to the World Health Organization (WHO). It affects 280 million people worldwide and is a leading cause of disability and financial loss. Patients with MDD present immunoendocrine alterations like cortisol resistance and inflammation, which are associated with alterations in neurotransmitter metabolism. There are currently numerous therapeutic options for patients with MDD; however, some studies suggest a high rate of therapeutic failure. There are multiple hypotheses explaining the pathophysiological mechanisms of MDD, in which several systems are involved, including the neuroendocrine and immune systems. In recent years, inflammation has become an important target for the development of new therapeutic options. Extracellular monomeric ubiquitin (emUb) is a molecule that has been shown to have immunomodulatory properties through several mechanisms including cholinergic modulation and the generation of regulatory T cells. In this perspective article, we highlight the influence of the inflammatory response in MDD. In addition, we review and discuss the evidence for the use of emUb contained in Transferon as a concomitant treatment with selective serotonin reuptake inhibitors (SSRIs).

## 1. Introduction

Major depressive disorder (MDD) is a mood disorder that has become a global health emergency, according to the World Health Organization (WHO), as it affects 280 million people around the world. It is the leading cause of disability and causes an excessive overall economic burden on health and social care [1]. Symptoms of MDD include irritability, feelings of profound sadness, and emptiness, all persisting for at least two weeks. Patients may experience a loss of pleasure in previously enjoyed activities, poor concentration, feelings of excessive guilt, low self-esteem, hopelessness about the future, thoughts about dying or suicide, disrupted sleep, changes in appetite or weight, and a general feeling of exhaustion or low energy [1,2].

An intricate combination of social, psychological, genetic, biological, and environmental factors may cause MDD [3]. People who have experienced adversity in their lives are more susceptible to developing depression [1]. MDD is linked to and influenced by deteriorated health due to cardiovascular disease, cancer, diabetes, chronic infections, and respiratory diseases [4]. Likewise, MDD can cause cardiovascular, metabolic, or immunological complications [5,6,7].

MDD is the second leading cause of disability worldwide, as described in the Global Burden of Disease Study [8]. This is aggravated by the fact that between 76% and 85% of patients do not receive treatment due to stigma, ignorance, or inaccessibility to mental health care [9]. It was estimated that during the 2010–2018 period, the monetary burden on the US government for adult depression cases was increased by 37.9% [10]. According to an economic analysis, costs resulting from MDD are estimated at approximately $382.4 billion, of which nearly two-thirds of the economic burden of MDD is attributed to indirect costs [11], such as absenteeism in the workplace, unemployment, all-cause mortality, and disability [10,11].

The treatment for MDD is fundamentally based on psychotherapy and pharmacotherapy, and is usually complemented with diet, exercise, etc. [12]. Several treatment guidelines for MDD suggest that moderate to severe depressive episodes should be treated with pharmacotherapy or a combination of pharmacotherapy and psychotherapy [12,13]. Mild MDD may initially be treated solely with psychotherapy; nonetheless, it is important to always take into consideration patient preferences and their previous treatment history [2,13]. Additionally, an initial conservative strategy of vigilance and non-pharmacological treatment may also be chosen for mild MDD [13].

Several drugs from different groups have been used as pharmacotherapy in patients with MDD; some examples are shown in Table 1. In a general sense, the effects of these drugs are based on improving mood and increasing motivation [14]. Antidepressants are often used in combination with psychological treatment and lifestyle changes [1,2]; they must be taken daily, and usually start to take effect in 2–4 weeks [1,2,14] This treatment ideally should be continued for four to nine months, and the duration of medication will depend on many factors, such as the symptoms and the patient’s risk of developing another depressive episode [12]. In some cases, antidepressants must be prescribed for years to prevent future relapses [1,12]. In this context, the Sequenced Treatment Alternatives to Relieve Depression (STAR*D) study, a large-scale efficacy trial, reported that up to 50% of patients with MDD required additional treatment to first-line treatment and approximately 30% of patients failed to remit even after four sequential therapies, suggesting a high prevalence of treatment resistance [15,16]. In another study, 15% of patients with MDD failed to remit, and 35% had multiple episodes during 23 years of follow-up; recurrence rates ranged from 40% to 85% [17]. MDD frequently occurs comorbidly with diabetes and cardiovascular disease, which is a prototypical example of physical–mental comorbidity. This interaction can exacerbate both conditions, as well as increase the rate of therapeutic failure [18,19,20]. Other factors that contribute to therapeutic failure are poor quality of life, increased symptomatology, polypharmacy, lower adherence to self-care regimens, increased risk for developing functional impairment, higher morbidity and mortality, and higher medical costs [18].

The high relapse rate observed in patients with MDD generates the need to explore new therapeutic alternatives. In this manuscript, we will review some theories about the pathophysiology of MDD, focusing on inflammation. As will be addressed below, inflammation has become relevant in the pathophysiology of MDD, so it may be a therapeutic target. Finally, the possibility of ubiquitin as a potential therapeutic agent in MDD will be explored due to its immunomodulatory capacity.

## 2. Neuroimmunoendocrine and Inflammation Alterations in MDD

The pathophysiology of major depressive disorder (MDD) involves a complex interplay between various systems, including the immune, nervous, and endocrine systems. In recent years, several hypotheses have been proposed to explain the pathophysiology of MDD, as described below.

### 2.1. Hypothesis of MDD Pathophysiology

Stress is one of the most important factors in the development of MDD, particularly stress in early life and chronic stress in susceptible individuals [21]. The pathophysiology of MDD is characterized by monoamine depletion, glucocorticoid receptor (GR) resistance, elevated levels of corticotropin-releasing hormone (CRH) and cortisol, and an overstimulation of glutamate receptors [22,23].

**Figure 1:**
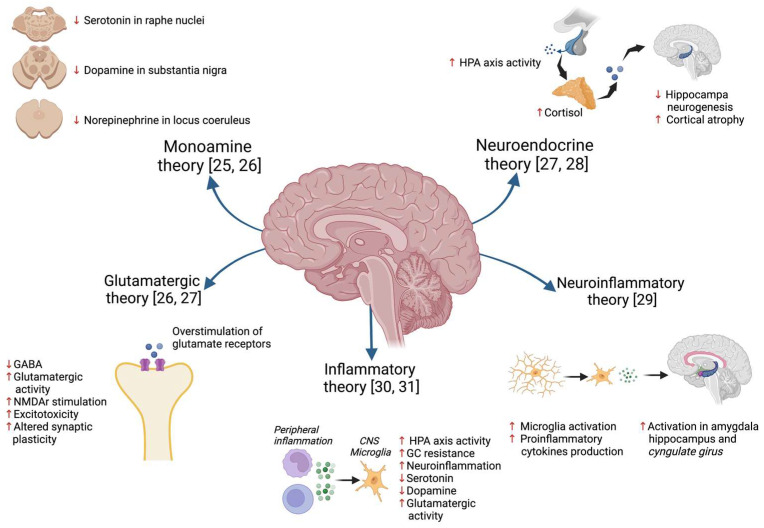
A description of some of the hypotheses that have been proposed to explain the pathophysiology of depression [25,26,27,28,29,30,31].

Several hypotheses have been proposed over time to explain the pathophysiology of MDD [24] (Figure 1), such as the classic “monoamine hypothesis”, which proposes that MDD is the result of the selective depletion of monoamines (serotonin, dopamine, and noradrenaline) in several areas of the central nervous system (CNS) [25,26]. Another theory is the “glutamate hypothesis”, which postulates that this neurotransmitter is also relevant in the formation of depressive symptoms and cognitive impairment through overstimulation of NMDA (N-methyl-D-aspartate) glutamate receptors, alteration of the AMPA (α-amino-3-hydroxy-5-methyl-4-isoxazolepropionic acid) receptor, altered glutamate reuptake, and dysfunction of the endocannabinoid system [27]. Another theory that has been put forward is the “neuroendocrine hypothesis” involving hyperactivity of the hypothalamic–pituitary–adrenal (HPA) axis, with an increase in cortisol production and a consequent reduction in hippocampal neurogenesis and plasticity in the cortex [27,28]. Recently, the neuroinflammation hypothesis has emphasized the mechanisms of microglial activation and the consequent production of proinflammatory cytokines, which in turn causes the hyperactivation of areas of the limbic system such as the amygdala, hippocampus, and cingulate gyrus, as well as metabolic alterations in the production of neurotransmitters induced by the activation of the enzyme indolamine-2,3-dioxygenase [29]. All of these theories described above attempt to explain MDD pathophysiology and symptoms, however, the inflammatory theory has become increasingly important and has been proposed as a central element in the pathophysiology of MDD [30,31]. One of the first pieces of evidence linking inflammation and behavioral changes was reported when immunotherapy with interferon (IFN)-α was introduced as a treatment for hepatitis C, in which the presence of depressive symptoms and even suicidal ideation was observed in patients receiving IFN-α treatment, as well as some reports of cognitive alterations and alterations in EEG patterns [32,33].

According to the results of several studies in human and animal models, cytokines have a neuromodulator function and induce mood disorders like depression or anxiety and cognitive impairments, such as memory loss. Table 2 shows examples of psychiatric effects induced by cytokines [34]. Studies in murine models and in humans have demonstrated that proinflammatory cytokines modify the metabolism of neurotransmitters such as serotonin and dopamine in brain regions such as the hypothalamus, locus coeruleus, and central amygdala [35,36]. Additionally, it has been shown that an increase in serum proinflammatory cytokines induces sickness behavior, characterized by a febrile response, anorexia, lack of motivation, social deprivation, and reduced movement [37,38]. Additionally, it has been documented that during systemic or chronic inflammatory diseases (e.g., chronic infections, autoimmune diseases, or chronic degenerative diseases) there is an increase in circulating proinflammatory cytokines that stimulate the brain and cause anxiety, anhedonia, social withdrawal, fatigue, and sleep disturbances [38,39,40].

Regarding the neuromodulator function of other cytokines, it has been described in murine models that IL-4 deficiency can lead to spatial learning and memory deficits [41]. Similarly, impaired short-term memory in a Y-maze test has been observed in IL-17A-deficient mice [42]. Both IL-4 and IL-17A mediate these effects by inducing the production of the brain-derived neurotrophic factor (BDNF), which promotes neurogenesis and is important in promoting learning behaviors [42,43]. Additionally, in a murine model, it was observed that the injection of IL-17A into the brain during the fetal stage of mice induces a significant impairment of social interaction in adulthood [44]. Finally, interferon-γ (IFN-γ) has been reported to regulate social behavioral brain connections. In several study models, it was observed that the inhibition of IFN-γ receptors or the STAT1 pathway in the prefrontal cortex caused a decrease in social interaction [45].

Cytokines are not the only component of the immune system involved in the development of psychiatric disorders. The complement system also regulates neurobiological processes such as neurogenesis, cell migration in the cerebral and cerebellar cortex, and synaptic pruning [46]. It was recently reported that the complement system protein C3 regulates conditioned fear behaviors in mice, while C3aR modulates unconditioned or innate anxiety behaviors [47]. Other complement molecules involved in the pathophysiology of psychiatric disorders have been described, such as the C1-inhibitor or C4A in schizophrenia [48,49].

### 2.2. HPA Axis and Glucocorticoid Resistance

**Figure 2:**
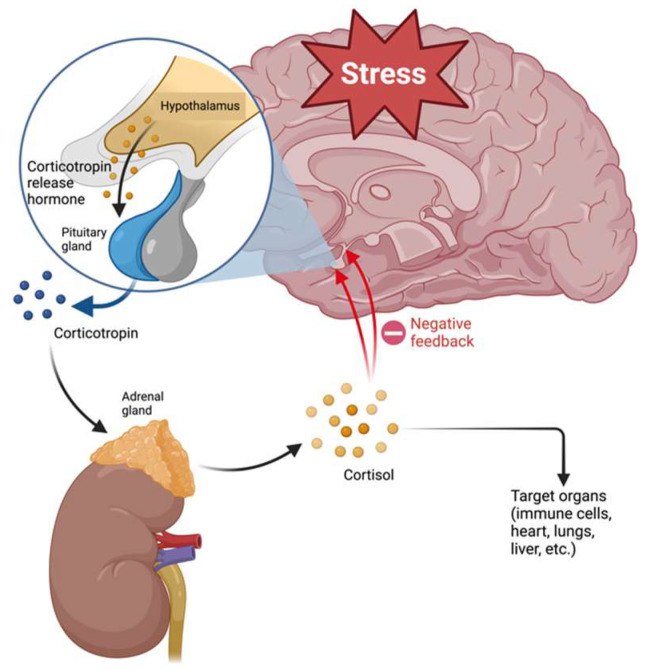
Regulation of hypothalamus–pituitary–adrenal axis.

As mentioned above, one of the hypotheses proposed to explain the pathophysiology of MDD involves alterations of the HPA axis. The hypothalamus–pituitary–adrenal (HPA) axis connects the nervous and endocrine systems and is formed by the hypothalamus, pituitary, and adrenal glands [50]. The HPA axis regulates the stress response by releasing CRH by the hypothalamus, which promotes corticotropin (ACTH) release by the pituitary gland. ACTH stimulates the adrenal glands to release cortisol, triggering the “fight or flight” response. This axis has a negative feedback regulation system that mediates the production of glucocorticoids (Figure 2) [51]. Activation of the HPA axis regulates different processes such as digestion, immune responses, mood, emotions, and energy metabolism [50].

**Figure 3:**
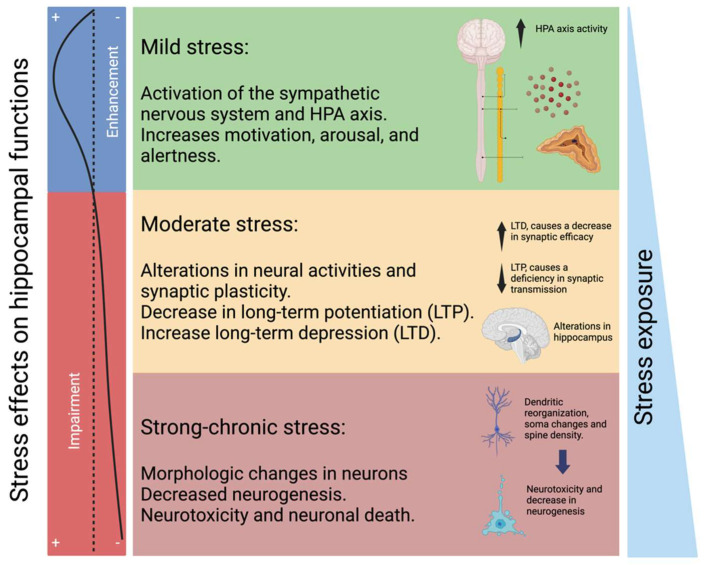
Changes in the hippocampus according to the magnitude of stress. Mild stress conditions are associated with short-term neurochemical alterations through the sympathetic nervous system, affecting motivational, arousal, and alertness functions. Moderate stress induces relatively more prolonged alterations in neural activities and synaptic plasticity. Chronic and strong stress conditions can affect hippocampal morphology, neurogenesis, and neurotoxicity. Mild stress can enhance hippocampal functions. Prolonged stress can induce alterations in hippocampal functions (left panel).

It has been reported that MDD patients have cortisol secretion disturbances, such as hyperactivity of the HPA axis or inversion of the circadian cycle in cortisol production, and it has been estimated that 90% of MDD patients have hypercortisolism [28,52]. Moreover, constant exposure to cortisol can lead to cognitive impairment, inducing irreversible changes in the neural system [53,54,55]. In this respect, hypercortisolism due to chronic stress affects the hippocampus, and it depends on stress duration. Consequently, if the stress lasts longer, there will be more damage to the hippocampus, ranging from modification of plasticity to neurotoxicity (Figure 3) [56,57].

Another feature observed in patients with MDD is glucocorticoid resistance, characterized by decreased sensitivity in glucocorticoid receptors such as cortisol receptors [58]. Several mechanisms have been proposed to explain glucocorticoid resistance, including alterations in glucocorticoid receptor (GR) function, changes in GR expression, alterations in glucocorticoid bioavailability through modification of serum protein binding, deficiencies in HPA axis feedback, and immune system inhibition [59,60]. It is worth mentioning that proinflammatory cytokines can also give feedback to the hypothalamus and anterior pituitary and thus increase HPA axis activity through modulation of GR function and expression [61]. It is well known that cortisol inhibits the immune response by inhibiting the expression of transcription factors such as NF-κB or decreasing the expression of adhesion molecules for diapedesis [62]. High levels of circulating glucocorticoids have been observed in patients with MDD and may coexist with elevated levels of proinflammatory cytokines such as IL-1β, IL-6, and TNF-α [58,61,63]. The simultaneous presence of elevated levels of glucocorticoids and cytokines in patients with MDD creates a complex interaction between the immune system and the HPA axis, in which a paradoxical phenomenon is observed in leukocytes. For instance, monocytes can generate proinflammatory cytokines but are ineffective in eliminating pathogens [64,65]. To explain this phenomenon, several studies report that elevated glucocorticoid levels in MDD induce glucocorticoid resistance, resulting in the inadequate regulation of the HPA axis, and this glucocorticoid resistance favors proinflammatory signaling pathways that are not inhibited by cortisol as normally occurs in the negative feedback loop [63,66]. A study in mice reported that chronic stress induces an increase in sympathetic activity in the bone marrow, which promotes the generation of Ly6C monocytes characterized by their resistance to glucocorticoids and their increased inflammatory capacity, as well as the production of more reactive oxygen species [64].

Some authors have considered that the normalization of HPA axis activity in patients with MDD may be a target for developing new therapeutic strategies [50]. This is an advantage since antidepressants such as SSRIs only reduce HPA axis hyperactivity by 30% after 52 weeks of treatment [67].

### 2.3. Proinflammatory Cytokines and Neurotransmitter Metabolism

**Figure 4:**
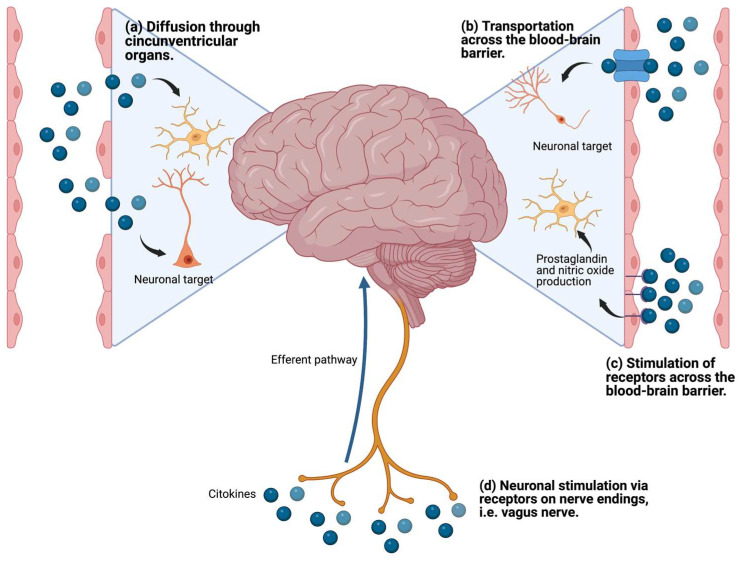
Mechanisms of communication of peripheral proinflammatory cytokines with the brain.

Cytokines are glycoproteins that act as messenger molecules to regulate inflammation and other cellular functions [68]. Cytokines are produced by different immune cells, parenchymal cells, endothelial and epithelial cells, fibroblasts, adipocytes, and stromal cells [68]. In addition, microglia, astrocytes, and neurons in the brain also produce cytokines. Several mechanisms by which peripheral cytokines stimulate the brain have been reported [69,70]: (i) by stimulating receptors in the blood–brain barrier (BBB) and producing metabolites in the brain; (ii) by accessing the brain through the circumventricular organs; (iii) by being carried through transporters in the BBB; and (iv) through stimulation of afferent fibers of the vagus nerve (Figure 4).

**Figure 5:**
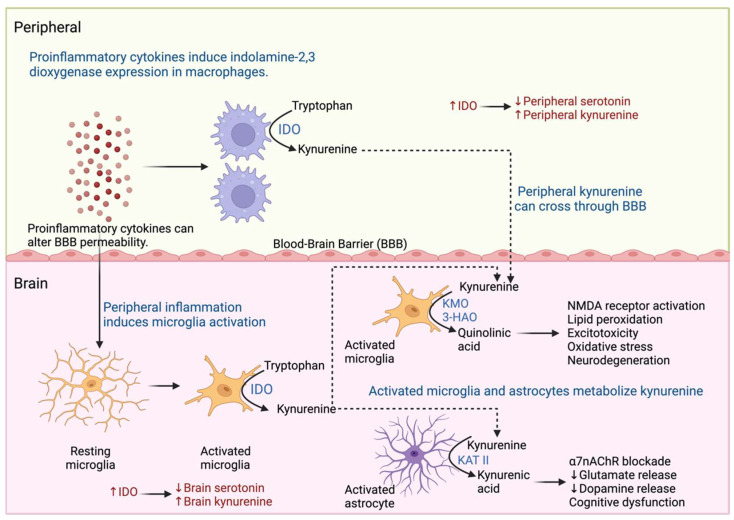
Kynurenine pathway in MDD. Inflammation promotes the generation of metabolites of the kynurenine pathway in the periphery and brain. Macrophages and microglia express indolamine 2,3-dioxygenase (IDO) and metabolize tryptophan to kynurenine. Kynurenine is metabolized by activated astrocytes by kynurenine aminotransferase II (KAT II) and activates microglia by kynurenine 3-monooxygenase (KMO) and 3-hydroxy anthranilic acid oxygenase (3-HAO), which generate neurotoxic metabolites that cause neurodegeneration and cognitive dysfunction.

Inflammation can be triggered in response to stressors such as infection, injury, surgery, and social stress, causing increased production of inflammatory mediators such as cytokines; the intensity and duration of the stimulus determines the level of inflammatory mediators release and the effects they cause [71]. As described above, proinflammatory cytokines produced in the periphery can stimulate the brain and generate an inflammatory response caused by the activation of neurons, microglia, and astrocytes [72]. During acute and chronic stress, proinflammatory cytokines induce changes in the metabolism of tryptophan, a precursor of serotonin, in the periphery and brain [73]. One of the mechanisms involved in the activation of indolamine 2,3-dioxygenase (IDO) is found in macrophages and microglia cells. IDO promotes tryptophan metabolism to the kynurenine pathway, resulting in decreased serotonin levels in the periphery and brain [74,75]. In addition, kynurenine produced in the periphery can cross the BBB and be metabolized by kynurenine produced in the brain by activated astrocytes and microglia [76]. Kynurenine is metabolized by astrocytes by kynurenine aminotransferase II (KAT II) and generates quinolinic acid which causes decreased production of dopamine and glutamate, as well as a blockade of α7nAChR cholinergic receptors, which is associated with cognitive dysfunction [76,77]. Similarly, activated microglia metabolizes kynurenine by kynurenine 3-monooxygenase (KMO) and 3-hydroxy anthranilic acid oxygenase (3-HAO) generates kynurenic acid, which stimulates NMDA receptors and causes lipid peroxidation, oxidative stress, excitotoxicity, and neurodegeneration [76,77] (Figure 5). In addition, it has been reported that chronic stress induces a decrease in serotonergic system function with an increase in the expression of SERT and p11 in peripheral blood mononuclear cells (PBMCs) [78]. On the other hand, the oxidative stress response caused during inflammation inhibits the production of tetrahydrobiopterin, a cofactor necessary for the activity of aromatic hydroxylase enzymes, which participate in the synthesis of neurotransmitters such as serotonin, dopamine, and norepinephrine, so that their inhibition causes a deficit in the production of monoamines [76,79]. These metabolic changes are related to the development of sickness behavior in patients with systemic inflammatory responses caused by injury or infection [38]. However, as soon as the inflammation is corrected the symptoms subside [38]. In cases of chronic stress, as in MDD, the above-mentioned alterations in the HPA axis such as glucocorticoid resistance, cause dysregulation of the inflammatory response and a low-grade chronic inflammation state, which generates a dopamine and serotonin deficit [80].

In the last two decades, there has been increasing evidence that MDD is associated with systemic immune system activation, reflected in inflammatory markers, immune cell numbers, and antibody titers [81]. Several meta-analyses have demonstrated that patients with MDD have higher levels of proinflammatory cytokines such as IL-1, IL-6, IL-12, IFN-γ, and TNF-α, and inflammatory mediators such as the circulating C-reactive protein [82,83,84,85,86,87]. Several studies that have evaluated cytokine concentration in MDD patients have shown that MDD patients frequently have higher levels of IL-6 and TNF-α compared to healthy volunteers [82,86]. However, in studies that quantify Th1 and Th2 cytokines in patients with MDD, there is a discrepancy regarding which cytokines are elevated. While some studies have shown an increase in Th1 cytokines, others have shown an increased concentration of Th2 cytokines [88,89,90,91]. These differences in results may be explained by the characteristics of the population studied, the heterogeneity of the pharmacological and nonpharmacological interventions, as well as the comorbidities of the individuals who participated in the study.

## 3. Inflammation as a Therapeutic Target for MDD

In recent years, an emphasis has been placed on the multidisciplinary treatment of patients with MDD [12]. The importance and benefits of physical activity and an adequate diet have been highlighted [92,93]. As discussed above, the pathophysiology of MDD is complex, and one of the points that should be underscored is inflammation. Several reports about the relationship of the brain–gut axis and microbiota with MDD have recently been published [93,94]. In this respect, it is important to note that gut microbiota is regulated by serotonin production, as well as T-regulatory cells and the cytokines they release [95,96]. It has been observed that patients with MDD have dysbiosis, which causes the production of proinflammatory cytokines and, consequently, the activation of Th1 and Th17 cells [97,98]. Furthermore, there is evidence of an elevation in HPA axis activation and cortisol release, which induces a decrease in serotonin released in the intestine and an increase in intestinal permeability [99]. Recent evidence proposes that increased intestinal permeability promotes the development of chronic low-grade inflammation through dysbiosis and the translocation of intestinal bacteria [100]. In addition to the production of proinflammatory cytokines, the normal levels of gut microbiota metabolites such as propionate, butyrate, and acetate are diminished, and there is also a decrease in serotonin production [101]. These changes have been related to the symptoms observed in MDD [99,101]. This is why dietary interventions have become crucial in managing MDD patients. Moreover, a key link has been established between physical activity and a decrease in proinflammatory cytokines in conjunction with a contribution to proper hippocampal function and, as a result, the down-regulation of the production of stress hormones such as cortisol [102,103].

**Figure 6:**
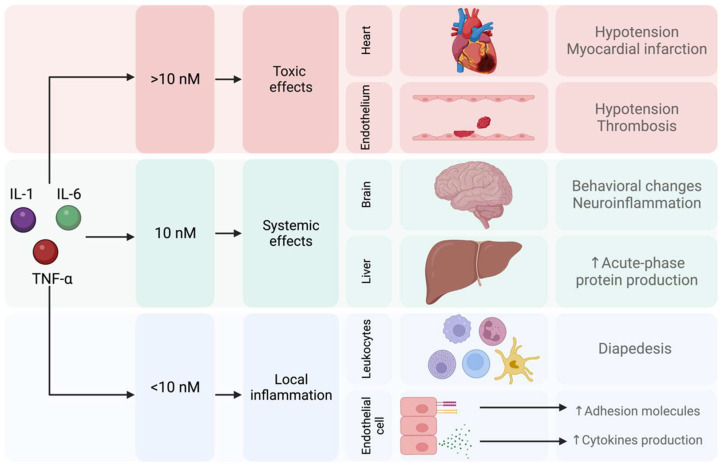
Local, systemic, and toxic effects induced by proinflammatory cytokines.

As mentioned above, inflammation plays an important role in the pathophysiology of MDD, and cytokines such as IL-1, IL-6, or TNF-α are involved. Subsequently, several mechanisms for low chronic inflammation have been investigated as new alternatives for treating MDD [104,105]. Proinflammatory cytokines have dose-dependent effects; it has been reported that a concentration below 10 nM triggers a local inflammatory response. Reaching a concentration of 10 nM of proinflammatory cytokines generates systemic effects that affect the brain and trigger behavioral changes such as sickness behavior. Finally, a concentration of proinflammatory cytokines higher than 10 nM is associated with toxic effects on the heart and endothelium [106] (Figure 6).

In addition, it is well known that the inflammatory response has intrinsic regulatory mechanisms, such as the production of anti-inflammatory cytokines by regulatory cells [107]. However, other regulatory mechanisms, such as cortisol release or the cholinergic anti-inflammatory pathway (CAP) that regulate inflammation, have been targeted as new therapeutic options [104,105].

### 3.1. Cholinergic Anti-Inflammatory Pathway and Inflammatory Reflex

**Figure 7:**
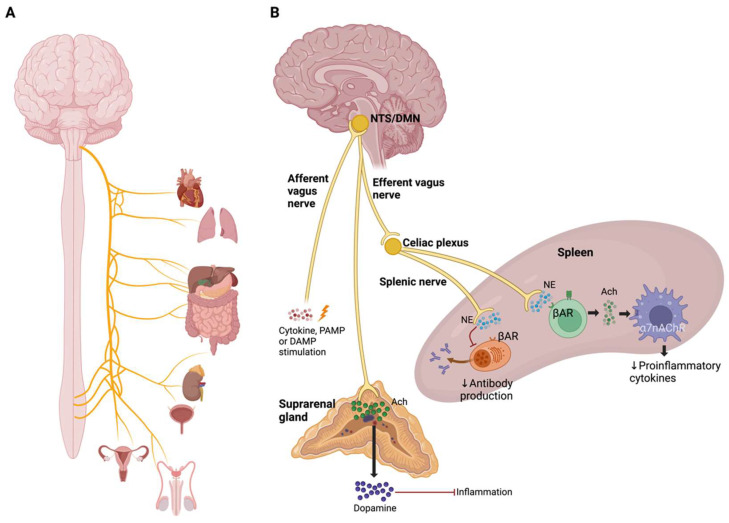
Cholinergic anti-inflammatory pathway. (**A**) Organs that are innervated by the vagus nerve. (**B**) Mechanisms of the cholinergic anti-inflammatory pathway. Afferent stimulation of the vagus nerve by cytokines, PAMPs, or DAMPs integrates into the nucleus of the solitary tract (NTS), which triggers an efferent response from the dorsal motor nucleus of the vagus nerve (DMN) to the spleen and the adrenal gland.

Acetylcholine (Ach) down-regulates the inflammatory response by suppressing the production of proinflammatory cytokines in peripheral immune cells through α7 nicotinic acetylcholine receptors (α7nAchRs) [108]. Cholinergic and catecholaminergic signals regulate lymphocyte activation in chronic inflammatory and autoimmune diseases [109]. CAP is a neural mechanism that inhibits the release of proinflammatory cytokines via afferent signaling by the vagus nerve and α7 receptors [110]. The CAP begins with afferent signaling from the vagus nerve, activated by cytokines, PAMPs, or DAMPs which, in turn, stimulate the nucleus of the solitary tract. Afterward, vagus efferent signals descend to the spleen to regulate the production of proinflammatory cytokines through Ach released by splenic T lymphocytes and dopamine released by the suprarenal gland (Figure 7) [110,111].

Vagus nerve stimulation increases Ach release by choline acetyltransferase (ChAT) in T cells. This Ach released interacts with α7nAChR expressed by macrophages resulting in the inhibition of NF-κB translocation, JAK2-STAT3 pathway activation and inflammasome activation, and, in consequence, causes suppression of proinflammatory cytokine production [109].

Norepinephrine and epinephrine inhibit the production of proinflammatory cytokines by stimulating β2-adrenoceptors, and both also stimulate the synthesis of anti-inflammatory cytokines [112,113]. However, it is also described that epinephrine has pro-inflammatory effects by promoting cytokine production in macrophages in a dose-dependent manner [114,115].

### 3.2. Extracellular Monomeric Ubiquitin (emUb)

In the 1940’s, it was described a process to obtain dialyzable leucocyte extracts (DLEs) from leukocyte lysate. Initially, DLE was described as a compound of low molecular weight peptides [116,117]. Subsequently, a standardized method to produce DLE from dialysis of human dialyzable leucocyte extracts (hDLEs) was developed in the National Polytechnic Institute in Mexico City, denominated “Transferon Oral^®^” [118]. This hDLE Transferon is considered a complex drug (CD). 

CDs are drugs in which the active pharmaceutical ingredient (API) is a heterogeneous substance composed of different but closely related molecules and, owing to its complexity, its mechanism of action (MOA), and its pharmacokinetic profile (PK) cannot be fully characterized [119]. The API of this immunomodulator is a mixture of human peptides with a molecular weight (MW) lower than 10 kDa, which is highly reproducible among batches, as described by extensive physicochemical and biological characterization [120,121,122,123].

Recently, a peptidome analysis of Transferon Oral^®^ was performed, and ubiquitin (Ub) was identified as one of its main components. This immunomodulator has a peptide concentration of 2 mg/5 mL, of which 1 μg is monomeric ubiquitin. The efficacy of this immunomodulator has been demonstrated in an HSV-1 infection assay in mice, showing improved survival in hDLE-Transferon treated mice when orally administrated. A similar assay using deubiquitinized Transferon (Ub was removed with an anti-ubiquitin antibody) showed protection from HSV-1 death, although less than whole hDLE-Transferon and monomeric Ub. These results suggest that the other components of this immunomodulator found in lower concentrations also have activity on the immune system [118].

Ub is a small protein weighing 8.5 kDa that is highly conserved in eukaryotic cells. Ub performs its functions in conjugation with a myriad of proteins [124]. Ub has intracellular and extracellular functions; intracellular functions are carried out by the binding of Ub to different protein substrates, generating a monoubiquitinization to which more Ub can be attached depending on the process. Ubiquitination is involved in diverse cellular processes such as apoptosis, organelle biogenesis, cell cycle, transcription and DNA repair, ribosome regulation, modulation of cell surface receptors, ion channels, and secretory pathways [125]. The extracellular functions of Ub are carried out by a monomeric form of Ub (emUb), which is found in serum, cerebrospinal fluid, lung, alveolar lining fluid, and urine. It has been described that emUb regulates different processes such as cell differentiation and development, immune response and inflammation, muscle and neuronal degeneration, morphogenesis of neuronal networks, and response to stress and to extracellular modulators [126]. mUb is a highly stable small protein whose intracellular activities are related to protein recycling, the modulation of NF-κB, and DNA repair [127,128]. However, many questions remain about Ub when it is found extracellularly due to cellular physiological processes or some event involving cell rupture [126]. The relevance of emUb first attracted the attention of researchers when a correlation was observed between high levels of serum ubiquitin and an increase in the survival rate in sepsis and severe burn injury patients; subsequently, increased levels of emUb were also observed in inflammatory diseases [129,130,131]. In preclinical models, the intravenous administration of emUb reduces TNF-α plasma levels and the mortality induced by endotoxin in pigs, whereas, in a lung polytrauma pig model, it decreases IL-8, IL-10, TNF-α, and CXCL12 in the damaged organ [132,133]. In addition, in PBMC exposed to lipopolysaccharide (LPS), emUb prevents the increment of pro-inflammatory cytokine TNF-α [130].

The hDLE Transferon Oral has been demonstrated to increase IFN-γ levels and CD4+ cells in people with herpes zoster, reducing refractory pain compared to patients who only receive acyclovir [134], which suggests that this immunomodulator could be used in diseases with inflammatory imbalances. In a herpes simplex virus type I (HSV-1)-infected mouse model, it was observed that the hDLE increases IFN-γ and reduces TNF-α and IL-6, improving survival [135]. Furthermore, the effect of the subcutaneous administration of the immunomodulator on the conventional pharmacological treatment of puppies infected with the Canine Parvovirus (CPV) type II was evaluated. The use of this immunomodulator increased the CPV-infected puppies’ survival rate (∆ 40%), decreased the circulating neutrophils and lymphocyte levels, and reduced cortisol and epinephrine concentrations during the critical period of the infection compared to puppies treated with conventional drugs alone [136,137].

**Figure 8:**
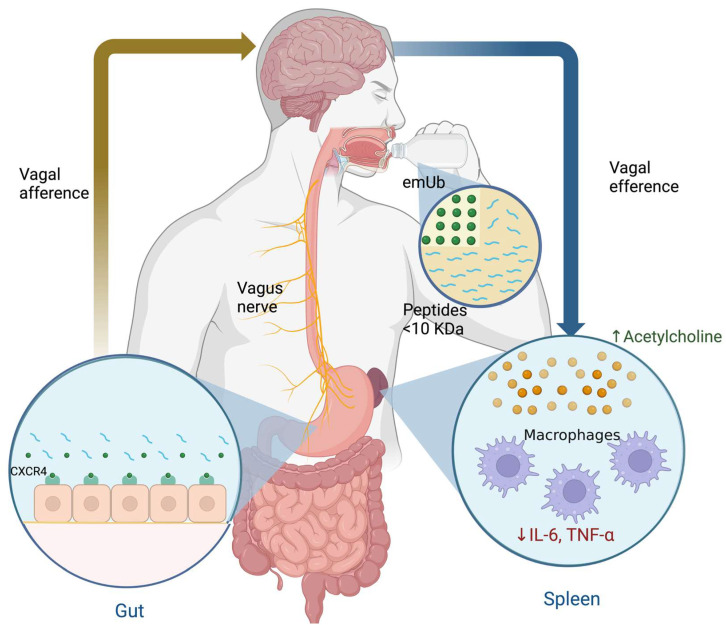
Hypothesis on the immunomodulatory effects of extracellular monomeric ubiquitin (emUb) as one of the main low molecular weight peptide components of human dialyzable leucocyte extract in patients with major depressive disorder.

The mechanism of action (MOA) of emUb is not fully understood, but experimental data suggest that Ub blocks partially the CXCR4/CXCL12 axis [126,138,139,140]. CXCR4 is related to the production of TNF-α in macrophages by the MAPK and NF-κB signaling pathways [141]. It has been described that Ub is a CXCR4 partial agonist which is expressed on various tissues such as nerves and leucocytes [139]. In this sense, it was suggested that emUb may interacts with the CXCR4 expressed in the afferent endings of the vagus nerve in the stomach after oral administration and, subsequently, causes a slight activation of the CAP (as mentioned above). The CAP induces the inhibition of TNF-α release by spleen macrophages through ACh signaling pathways and decreases serum cortisol concentrations (Figure 8) [118,142,143]. In addition, emUb has been shown to induce immunomodulatory effects in macrophages and lymphocytes, such as decreased chemotaxis, proliferation, and cytokine secretion through CXCR4 receptor-mediated regulation of the inflammatory response triggered by damage-associated molecular patterns (DAMPs) and pathogen-associated molecular patterns (PAMPs), in order to avoid a hyper-inflammatory response [131,144]. A third possible immunomodulatory mechanism may involves professional antigen-presenting cells like dendritic cells (DCs). The maturation of DCs is crucial for stimulating T-cell responses to foreign antigens and for maintaining immune tolerance to self antigens [145]. DC maturation requires the up-regulation of MHC class II (MHC-II) on the surface, as well as co-stimulatory molecules, such as CD80 and CD86 [145,146]. An important mechanism controlling surface expression of MHCII and CD86 is the ubiquitin-dependent degradation of these molecules [146,147]. Ubiquitination of MHC-II and CD86 is carried out by Membrane-Associated RING-CH-1 (MARCH1), which is an E3 ubiquitin ligase and promotes endocytosis and lysosomal degradation of MHC-II and CD86 [145,146,147]. Under basal conditions, MARCH1 is downregulated during DC maturation and IL-10 induces its expression. Thus, emUb administration reduces the surface expression of MHCII and CD86 on DCs and THP-1 macrophages [145,146,148]. Finally, it has been observed that ubiqutinization of MHC-II and CD86 promotes the differentiation of regulatory T cells [146].

## 4. Use of emUb as a Part of the Peptide Composition of the hDLE in MDD Treatment

Since previous evidence in animal models and in humans showed that hDLE-Transferon has immunoregulatory properties that cause a decrease in circulating proinflammatory cytokines and cortisol, in addition to elevating IFN-γ levels. For this reason, the use of this immunomodulator was evaluated as a complementary treatment to selective serotonin receptor inhibitors (SSRIs) in patients with MDD.

First, the immunological phenotype of patients with MDD was identified in healthy volunteers and MDD patients, in which were determined the proinflammatory cytokines concentration in serum and 24 h urine cortisol concentration, as well as T subclasses, B and NK lymphocyte. Results showed that subjects with MDD had higher cortisol and TNF-α levels, increased CD3+CD8+ and NK percentages, decreased B lymphocyte counts, and no significant variations in CD3+CD4+ lymphocytes when compared to healthy individuals (Table 3). Additionally, in the same study, it was also observed that patients with MDD had higher levels of IL-4 and IL-13 and significantly lower concentrations of IL-2 and IFN-γ [149].

Subsequently, a 52-week follow-up of MDD patients and healthy volunteers was conducted, treating MDD patients with selective serotonin receptor inhibitors (SSRIs) in order to evaluate the long-term effect of SSRI administration on cortisol concentration and pro/anti-inflammatory cytokine profile (Table 4). The Hamilton Depressive Rating Scale (HDRS) and the Beck Depression Inventory (BDI) were applied monthly, and levels of IL-1β, IL-10, IL-2, IFN-γ, IL-4, and IL-13, as well as 24 h urine cortisol were determined at weeks 0, 5, 20, 20, 36, and 52 of treatment. At week 20 of treatment, HDRS and BDI indicated a remission of the depressive episode concomitant with increases in IL-2 and IL-1β but no change in cortisol. Moreover, a significant reduction in cortisol levels, with an increase in IL-1β and IFN-γ and a decrease in Th2 cytokines, were observed towards the end of clinical follow-up at week 52 [67].

Finally, the administration of hDLE-Transferon (which contains emUB) as an adjuvant treatment to SSRIs in patients with MDD was evaluated. MDD patients received SSRIs or SSRIs plus hDLE. Serum concentrations of the proinflammatory cytokines IL-1β, IL-2, and IFN-γ; the anti-inflammatory cytokines IL-4, IL-13, and IL-10, as well as 24 h urine cortisol quantification was determined along 52 weeks of treatment (Table 5). After 52 weeks of treatment, it was detected a decrease of 30% of Urine cortisol in the SSRI-treated group, whereas in the SSRI plus hDLE-treated group urine cortisol was reduced by 54%. Cortisol reduction in patients treated with SSRI plus hDLE correlated with reduction of anti-inflammatory cytokines levels and increases levels of proinflammatory cytokines which suggests a decrease in HPA axis hyperactivity accompanied by a more physiological balance between pro and anti-inflammatory cytokines [150]. It is important to emphasize that patients treated with SSRI plus hDLE presented a higher IFN-γ concentration in comparison with patients that received SSRIs.

As mentioned above, the proposed mechanism of action of Transferon is an immunomodulatory effect acting on the CXCR4/CXCL12 axis and thus activating the CAP, which would generate the inhibition of proinflammatory cytokines released in the spleen through Ach release and decreased cortisol levels (Figure 8). Therefore, the effect of SSRI plus hDLE consumption in MDD patients may cause a significant decrease in the HPA axis activity and an increase in IFN-γ levels, which were better results than those seen in patients who only received SSRI treatment. 

It has been described that serotonin promotes IFN-γ production, so the difference in IFN-γ levels observed in patients treated with the SSRI plus hDLE combination suggests that this treatment offers a better response in MDD patients. However, further studies are needed to understand the molecular mechanisms that cause clinical and biochemical improvements [151,152]. Despite the observed results of the use of SSRIs plus hDLE in patients with MDD, several questions remain to be resolved about the MOA by which the emUb regulates inflammatory processes, alone or as part of the peptide component of this immunomodulator. However, the fact that both modulate cytokines such as TNF-α, IL-6, IFN-γ, and cortisol strongly suggests that these biotherapeutics have the potential to complement the treatment of patients with MDD. More clinical trials evaluating treatment with SSRIs plus hDLE are required, since one of the limitations of the results is the size of the population evaluated.

## 5. Conclusions

Evidence shows that patients with MDD have altered proinflammatory molecules in circulation, glucocorticoid resistance, and significant variations in neurotransmitter levels in the brain and periphery. According to several reports, there is a high rate of therapeutic failure in MDD, so new therapeutic options need to be explored, and inflammation has become a critical target. The combination of SSRIs with hDLE has shown immunomodulatory effects, due to a probable CAP activation through the partial agonism of the CXCR4/CXCL12 axis. As a consequence, the inflammatory and cortisol imbalance is improved, inducing an improvement clinical and molecular status of patients, which makes this treatment a promising therapeutical option. However, further studies are needed to fully understand the effector mechanisms by which emUb contained in hDLE generates immunoregulatory effects in MDD patients. In addition, further clinical trials are needed to validate the potential use of emUB as an adjunctive treatment in MDD. Physicians should consider inflammation as a fundamental part of MDD pathophysiology, so it is important to treat possible causes of inflammation involving dietary habits, lifestyle, addictions, among other factors. 

## Figures and Tables

**Table 1:** Types of antidepressants.

Category	Mechanism of Action	Examples
Selective serotonin reuptake inhibitors (SSRIs)	Inhibit serotonin reuptake, thus increasing serotonin activity.	Citalopram Escitalopram Paroxetine Sertraline Fluoxetine Fluvoxamine
Serotonin–norepinephrine reuptake inhibitors (SNRIs)	Block serotonin and norepinephrine reuptake in the synaptic button, increasing postsynaptic receptors’ stimulation	Venlafaxine Desvenlafaxine Duloxetine Milnacipran Levomilnacipran
Atypical antidepressants	This group is characterized by different mechanisms of action, with the following examples: Bupropion inhibits dopamine and norepinephrine reuptake. Mirtazapine blocks α-2 adrenergic receptors on the cell bodies and nerve terminals and increases the release of norepinephrine into the synapse. Mirtazapine works by blocking alpha-2 adrenergic receptors on the cell bodies and nerve terminals, promoting the release of norepinephrine into the synapse, and in addition antagonizes 5-HT receptors	Bupropion Mirtazapine Agomelatine
Serotonin modulators	This group has different mechanisms of action on the serotonergic system. Trazodone acts upon postsynaptic serotonin 5-HT2A and 5-HT2C receptors and weakly inhibits presynaptic serotonin reuptake. In addition, it has additional postsynaptic alpha-adrenergic receptors and histamine receptors blocking activity. In addition, it blocks α-adrenergic receptors and histamine receptors in the postsynaptic button. Nefazodone antagonizes postsynaptic serotonin 5-HT2A receptors and inhibits presynaptic serotonin and norepinephrine reuptake. Vortioxetine acts as a 5-HT1A receptor agonist and a 5-HT3 and 5-HT7 receptor antagonist.	Nefazodone Trazodone Vilazodone Vortioxetine
Tricyclic antidepressants (TCAs)	Inhibit the reuptake of norepinephrine and serotonin at the presynaptic neuronal membrane.	Amitriptyline Clomipramine Doxepin Imipramine Trimipramine Desipramine Nortriptyline Protriptyline Maprotiline Amoxapine
Monoamine oxidase inhibitors (MAOIs)	Inhibit the monoamine oxidase enzyme responsible for catabolizing serotonin, norepinephrine, and dopamine.	Selegiline Moclobemide Tranylcypromine Isocarboxazid Phenelzine

**Table 2:** Psychiatric effects induced by cytokines.

Cytokine	Effect
IFN-α	Fatigue, depression, thought disorders, psychosis and suicidal ideation, stress, anxiety, decreased substance P, myalgia, psychomotor retardation, anorexia, social isolation, irritability, and cognitive disorders (lack of concentration, memory impairment, and bradypsychia)
IFN-β	Fatigue, depression, and bradypsychia
IFN-γ	Modulates social behavior by regulating the connection of social interaction brain areas
TNF-α	Anorexia, fatigue, stress, upregulation of substance P expression, rapid eye movements during sleep, and increased release of excitatory neurotransmitters; noradrenaline and adrenaline stimulate its release
IL-1β	Somnolence, confusion, hallucinations, hyperalgesia, fatigue, fever, sleepiness, myalgia, and substance P antinociception (increased GABA and decreased NMDA); noradrenaline and adrenaline stimulate its release
IL-2	Confusion, delusions, depression, psychosis, myalgias, and cognitive dysfunction
IL-4	Regulates higher mental functions such as memory and learning
IL-6	Stress, fatigue, hyperalgesia, depression, and activation of the sympathetic nervous system; noradrenaline, adrenaline, and substance P stimulate their release
IL-8	Mediates sympathetic pain; substance P stimulates its production
IL-10	Blocks pain
IL-17A	Modulates anxiety through meningeal γδ T cells

**Table 3:** Variables evaluated in patients with MDD and healthy volunteers.

Variables	Effect
Cortisol	↑
T helper cells	ns
T cytotoxic cells	↑
NK cells	↑
B cells	↓
IL-1β	↓
TNF-α	↑
IL-6	ns
IL-2	↓
IFN-γ	↓
IL-4	↑
IL-13	↑

ns: indicates no significant difference. ↑: indicates higher levels. ↓: indicates lower levels.

**Table 4:** Variables measured during 52-week follow-up in patients with MDD treated with SSIRs.

Variables	Comparison of Patients with MDD vs. Healthy Volunteers		
	W0 vs. HV	W20 vs. HV	W52 vs. HV
Cortisol	↑	ns	↑
IL-1β	↓	↑	↑
IL-2	↓	ns	↓
IFN-γ	↓	ns	↑
IL-10	↑	↓	↓

HV: healthy volunteers. ns: indicates no significant difference. ↑: indicates higher levels. ↓: indicates lower levels. W: week of clinical follow-up.

**Table 5:** Variables were assessed at 52-week follow-up in patients with MDD treated with SSIR and SSIR + hDLE.

Variables	Comparison of Patients with MDD vs. Healthy Volunteers (HV)					
	W0		W20		W52	
	SSRI + hDLE vs. HV	SSRI + hDLE vs. SSRI	SSRI + hDLE vs. HV	SSRI + hDLE vs. SSRI	SSRI + hDLE vs. HV	SSRI + hDLE vs. SSRI
Cortisol	↑	ns	ns	↓	ns	↓
IL-1β	↓	ns	↑	ns	ns	↓
IL-2	↓	ns	ns	ns	ns	↑
IFN-γ	↓	ns	ns	↑	ns	↑
IL-10	↑	ns	ns	↑	ns	↑

ns: indicates no significant difference. ↑: indicates higher levels. ↓: indicates lower levels. hDLE: human dialyzable leucocyte extracts. HV: healthy volunteers. SSRI: selective serotonin reuptake inhibitors treatment. SSRI + Transferon: selective serotonin reuptake inhibitors plus extracellular monomeric ubiquitin in mix treatment. W: week of clinical follow-up.

## Data Availability

No new data were created or analyzed in this study.
